# What do they look for and what do they find? A coproduced qualitative study on young people's experiences of searching for mental health information online

**DOI:** 10.1111/papt.12550

**Published:** 2024-10-14

**Authors:** M. E. Loades, N. Higson‐Sweeney, B. Teague, J. Leas, C. Payne‐Cook, A. V. Slastikova, H. Peel, G. Chamberlain, L. Ferguson, K. Janes, T. Rhodes, E. C. Roupa, L. Biddle

**Affiliations:** ^1^ Department of Psychology University of Bath Bath UK; ^2^ NSFT Research Norfolk and Suffolk NHS Foundation Trust Norwich UK; ^3^ Department of Clinical Psychology and Psychological Sciences University of East Anglia, Norwich Research Park Norwich UK; ^4^ Population Health Sciences, Bristol Medical School University of Bristol Bristol UK

**Keywords:** adolescents, coproduction, depression symptoms, early help, mental health, online help‐seeking, qualitative, think aloud

## Abstract

**Background:**

Many young people (YP) struggle with their mental health and look online for help. To capitalise on their digital presence, we need to better understand how and where they seek information online and what they think of what they find.

**Method:**

We recruited 24 YP (aged 13–18 years). Online interviews were co‐conducted by research team members and trained young researchers. We presented a persona with depression symptoms and asked about potential sources of information/support they might seek. They were also asked to think aloud while searching online and reviewing mental health resources (NHS, Young Minds). We used reflexive thematic analysis.

**Results:**

Analysis generated four themes: (1) the online help‐seeking process, showcasing where YP look for information and why; (2) the mismatch between the information YP expected to find and the reality; (3) the strategies YP employed to determine a source's trust and credibility and (4) individual differences that can influence help‐seeking.

**Conclusion:**

Participants initiated their online search by Googling symptoms. They trusted NHS websites for basic medical information, while charities provided detailed content. Despite scepticism about content, social media offered validation. Online resources should prioritise visual appeal, user‐friendliness, age‐appropriate and personalised content and peer insights. Codesign is imperative to ensure high‐quality, impactful research.

## INTRODUCTION

Depression becomes more common during adolescence (Lu, 2019; Rapee et al., 2019) but the provision of timely help is lacking. By age 18, one in 10 young people (henceforth YP, used to refer to adolescents) will have clinically significant depression (Solmi et al., 2021), characterised by sadness and/or irritability and lack of enjoyment and/or motivation (American Psychiatric Association, 2013). Additionally, one in three YP have elevated depression symptoms at any point in time (Shorey et al., 2021). These symptoms, even if subthreshold (Carrellas et al., 2017; Crockett et al., 2020; Noyes et al., 2022), can significantly impact functioning. Evidence‐based treatments can help YP overcome depression (Thapar et al., 2022). Yet, many who need evidence‐based help struggle to access it due to limited service capacity and help‐seeking barriers (Pimenta et al., 2021; Radez et al., 2020; Valentine et al., 2024), like lack of knowledge (Thapar et al., 2012, 2016) and stigma (Aguirre Velasco et al., 2020; Patil et al., 2018). YP from minority groups who are more vulnerable to depression (Deighton et al., 2019; Lavner et al., 2021) are disproportionately affected by these barriers (Meechan et al., 2021). To improve timely access to help and support for YP who are beginning to experience depression symptoms, we need to make information and links to sources of support more accessible and developmentally appropriate on the platforms and media that YP already engage with (e.g. aligning sources of support with their preference for self‐reliance (Gaudreau et al., 2023)). However, we do not know where YP looks for help or what they think of the information they find.

The current generation of YP are ‘digital natives’ who spend a lot of time online. In 2020, 96% of UK households had Internet access (ONS, 2020), 83% of 12‐ to 15‐year‐olds owned a smartphone and 71% had a social media profile (OFCOM, 2020/21). The potential benefits of seeking information and help online include accessibility and anonymity, as well as the privacy and degree of control provided (Pretorius et al., 2019; Wong et al., 2021). However, some of the most frequently endorsed barriers to online help‐seeking include lacking trust in the Internet or in people online (including doubting credibility), lack of confidentiality and not feeling like they have an appropriate problem to seek help for (Barrow & Thomas, 2022; Best et al., 2016; Lipshits‐Braziler et al., 2020; Pretorius et al., 2019; van den Toren et al., 2019). It has been proposed that there are three potential functions of online information and support for help‐seekers, specifically: (1) providing information about their symptoms; (2) connecting them to others, like professionals or peers; and (3) as an alternative to other offline sources of help (Pretorius et al., 2019).

To know how best to provide information and support to YP, we need to understand where they look for help online and what they think of the information they find. In this study, what we mean by mental health help‐seeking is ‘….an adaptive coping process that is the attempt to obtain external assistance to deal with a mental health concern…’ (Rickwood & Thomas, 2012), and this process includes becoming aware of symptoms and the need for help, expressing this need, identifying sources of hep, and willingness to disclose difficulties (Rickwood et al., 2005). Thus, this process might include seeking information about symptoms and sources of help, including self‐help. The approach to providing the public with information about (mental) health has generally been to place this on websites. There is some conflicting evidence that YP do look at websites relating to their health. A systematic review of 28 studies examining online help‐seeking for mental health difficulties (published prior to August 2017) reported that the most common source of help sought was via Internet search engines, with YP also going directly to known government and charity websites, social media and social networking sites (Pretorius et al., 2019). One subsequent US‐based study found that the Internet was the most common source of information sought by YP, including young adults, prior to being diagnosed with depression or anxiety, and they tended to seek information about symptoms they were experiencing (Van Meter et al., 2019). However, other recent studies suggest that websites may not be the primary online source of information for all YP; for example, the most favoured way of seeking help online based on a sample of 525 YP recruited from schools in the United Kingdom was via private messaging on social networking sites (Lipshits‐Braziler et al., 2020). YP's reasons for using social networking sites include seeking social support (Meng et al., 2017) and expressing their distress and frustrations (Ophir, 2017; Vermeulen et al., 2018). Furthermore, YP do not necessarily use public health websites; even when instructed to look at a website to support treatment uptake, a study of YP found that only 50% did so (Radovic et al., 2018). Additionally, whilst some found the Internet helpful, others did not, and some had also posted on social media about their symptoms (Van Meter et al., 2019). Therefore, we need to better understand how and where YP seek information and support online, including early help for depression symptoms, to capitalise on combining their digital presence with early help messaging.

Despite the breadth of existing research, there remain many unanswered questions about where and how YP look for help online. When planning this study, our YP advisors and their supporters (parents/caregivers, teachers and GPs) highlighted how difficult it is to (1) ask for and get help at the point of need, before things escalate, and (2) know which of the many self‐help resources available are effective and trustworthy.

### Aims and objectives

We need to know how best to enable all YP to access early help in ways that are relevant, credible and available in the places they visit online. As such, in the current study, we aimed to explore where YP in the United Kingdom look for help online for low mood and what they think of what they find, using semi‐structured qualitative interviews co‐conducted with a trained young researcher. We also involved our trained young researchers throughout the analysis and sense‐making stages as well as at the design stage. We opted for using this participatory research approach to enhance the relevance and validity of our study (Warraitch et al., 2024), and we assumed that the presence of a young person interviewer would help to build trust with our participants and would be empowering. We focused on those aged 13–18 years as depression prevalence dramatically increases during this age range (Solmi et al., 2021), and YP are developmentally transitioning from being more dependent on adults to functioning more autonomously, including when seeking help and using the internet. We were interested in reaching all YP, irrespective of mental health status or history, and including those from underserved groups. Our specific research questions were:

1. Where do YP look for information when they start to experience symptoms of depression?
2. Where do they look for information via the Internet and social media, and do they use different platforms for different purposes?
3. How do they search for information and what are their experiences of what they find?

## METHOD

### Participants

**FIGURE 1:**
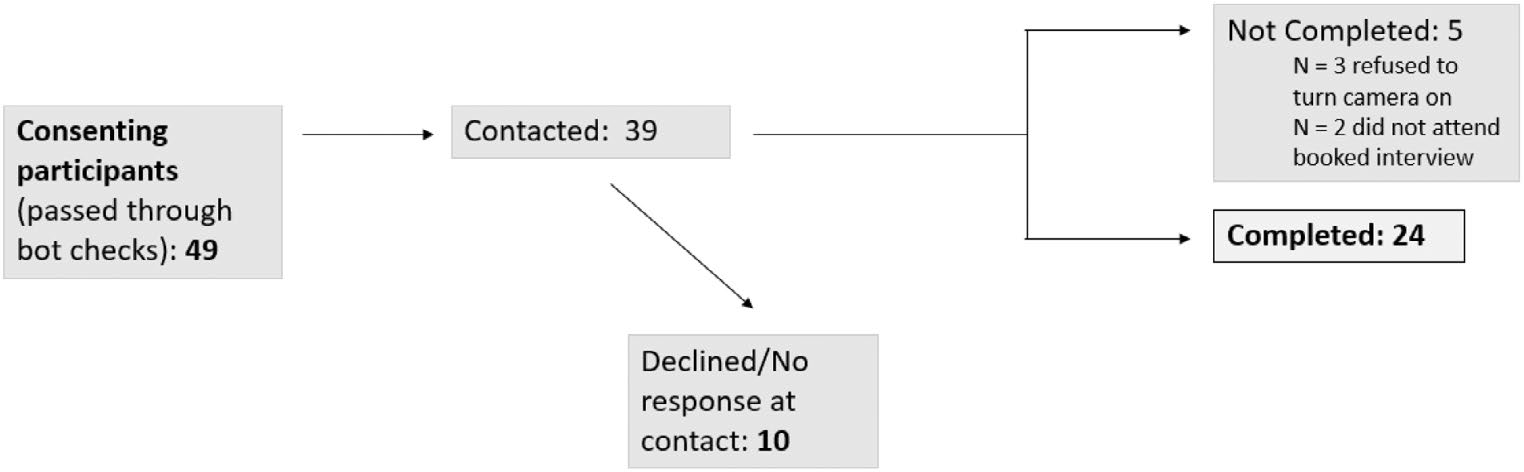
Recruitment flow chart.

A series of adverts were created with input from a Young Person's Advisory Group (YPAG; examples in supplementary materials S1). Participants were recruited via social media, including Instagram and X (formerly Twitter), and mailing lists, including via schools and community organisations that reach diverse groups (e.g. YP from lower income families, ethnic minorities and those who identify as lesbian, gay, bisexual, transgender and non‐binary, queer and questioning, intersex, asexual, aromantic, or agender and those who are part of that community, henceforth LGBTQIA+). We used convenience sampling, contacting all participants who opted in by completing a brief online contact form and passing through bot checks (see Figure 1 and supplementary materials S2). English‐speaking YP aged 13–18 years and residing in the United Kingdom were eligible to take part. They were not required to have experience of depression or help‐seeking. Participants were excluded if they did not meet eligibility criteria or were unable to complete study measures, which included switching on their camera for at least some of the online interview to validate their identity as a YP. Recruitment occurred during July to August 2023 to maximise availability during school holidays. Sample size was guided by the concept of information power (Malterud et al., 2016) (i.e. collecting sufficient information to answer our research questions in terms of depth and consistency of the data, as well as diversity of the sample).

### Procedure

We obtained ethical approval from the University of Bath's Psychology Research Ethics Committee (23‐057) and pre‐registered our study protocol on the Open Science Framework (https://osf.io/a6k7r). During recruitment (July–August 2023), interested potential participants could use a link/QR code to access an online screener to determine eligibility, followed by information about the study and a consent form. Participants aged ≥16 completed the consent form themselves, whereas those aged 13–15 years required parent/caregiver consent. Consenting/assenting participants were asked to provide their contact details and generate a memorable pseudonym for themselves. Brief demographic information was sought to monitor diversity and, if necessary, would have been used to purposively sample, however this was not required in practice.

Interviews were conducted and recorded online using Microsoft Teams. Participants were required to switch their camera on at the beginning of the interview for identity validation and were encouraged to keep it on for the duration of the interview. All participants did keep their cameras on, and all screens were shared when requested to do so. Twenty‐three interviews were conducted by a pair of researchers, one of whom was a salaried member of health research team from NSFT Research (*n* = 5), and one of whom was a young researcher (*n* = 4). One interview was conducted solely by an NSFT researcher due to scheduling issues. Both interviewers kept their cameras on throughout. Some questions on the topic guide were asked by the young researcher, and others by the research staff member. At the end of the interview, participants were given debrief information listing mental health resources and explaining how they could access post‐interview support. Participants were given a £20 Amazon e‐voucher as compensation for their time and effort.

### Young research team (YRT)

Based on a participatory research framework (Collins et al., 2018; Stacciarini et al., 2011), YP were involved as partners in co‐conducting the research. We recruited a team (YRT) of four secondary school students (aged 16–18) via mailing lists of YP who expressed interest in these roles. The YRT reviewed the topic guide, co‐conducted the semi‐structured interviews and were involved in the data collection (coding, theme generation and labelling) and write‐up. They are named co‐authors by choice. They were provided with 1.5 h training in conducting qualitative interviews, including confidentiality, interviewing techniques, safeguarding processes, self‐care and study‐specific processes. They were closely supervised throughout by NSFT Research, and at the end of the interview stage, they met with MEL and BT to reflect on their learning from the process. They were required to sign confidentiality agreements and were compensated £10/h for their time during the interview stage (with payment for preparation time) and £20/h (no additional payment for preparation time) for their time during the analysis stage. They were provided with an institutional email address, Microsoft Teams access and, where necessary, were lent an institutional laptop to support their involvement.

### Measures and materials

#### Demographics and clinical characteristics

We asked participants to self‐report basic demographic information, including age in years, sex assigned at birth, gender identity (including a list of terms; see supplementary materials S3 and a free text box to opt to self‐identify), ethnic group (UK census 2021 categories) and the 2‐item MacArthur Social Status Scale (Goodman et al., 2001) as an indicator of deprivation. Participants also completed the Patient Health Questionnaire‐2 (PHQ‐2) (Kroenke et al., 2003; Richardson et al., 2010), which is a 2‐item self‐report measure that assesses frequency of depressive symptoms, specifically low mood and anhedonia, over the past 2 weeks. Each item is rated on a 4‐point scale, from ‘not at all’ (0) to ‘nearly every day’ (3). Higher scores indicate greater depressive symptoms, and a score of ≥2 indicates at least moderate depressive symptoms (Pitts et al., 2023).

#### Semi‐structured interview topic guide

A flexible, semi‐structured interview topic guide (supplementary materials S4) was used to guide interviews. This was developed with input from members of the YPAG and YRT. In the first part of the interview, the YRT interviewer read out a ‘persona’ generated by the YRT. This persona (Sally) described a YP who was beginning to struggle with low mood and not enjoying activities they previously liked. Participants were then asked open‐ended questions by the YRT interviewer to explore where the persona might initially seek help, with probes for how they would search online and on social media, and what might influence their perceptions of the information they found. In the second part of the interview, led by the research team interviewer and using ‘think aloud’ techniques (Wolcott & Lobczowski, 2021), participants were then asked to share their screen (with support given to navigate this where needed) and to search the Internet for information about early help for depression, describing their in‐the‐moment perceptions of what they found and rating relevance, trustworthiness and credibility. Interviewers used probe questions where needed. In the third part, the interviewers then shared screenshots of a selection of websites (specifically an NHS Child and Adolescent Mental Health Service [CAMHS] website and Young Minds website; see supplementary materials S4) and asked participants about their thoughts, again probing various constructs (e.g. understandable, credible, personally relevant, important, useful, motivate action and likeable). The fourth part of the interview, led by the YRT interviewer, described online single‐session interventions and asked participants what they thought of these (this data was not explored in the current paper). Finally, the interview was closed by the research team interviewer, including debrief information and an explanation of next steps. Throughout, the interviewer pair supported one another and the conversation and added probes for each other's questions where necessary. Thus, the interaction between the interviewer pair was conversational and collaborative.

### Data analysis

An initial transcript was generated through Microsoft Teams, which was subsequently checked and corrected for accuracy and anonymised where necessary, from which point the participant's chosen pseudonym was used. Information from the screen share section of the interviews was captured by watching the interview recordings and noting in a Microsoft Excel spreadsheet what search terms participants entered into the search engine and what websites they chose to click on.

The demographic and clinical characteristics of the sample were analysed descriptively using Microsoft Excel. Reflexive thematic analysis (Braun & Clarke, 2006, 2013, 2019) was used to iteratively code the data and generate themes. This was primarily inductive (data‐driven) and undertaken at a semantic level. As a first step, members of the research team (MEL, NH‐S, JL and CP‐C) familiarised themselves with three transcripts, making free‐form notes of what was interesting and independently labelling chunks of text (codes), before convening to discuss and compare codes. Disagreements were resolved through dialogue and iteratively revisiting the transcripts. Going forward, this conversation was held in mind, but a codebook was not developed. Analysis then followed Braun and Clarke's (Braun & Clarke, 2006, 2019) six‐phase process, as follows. In phase one, transcripts were divided up between MEL, NH‐S, JL and CP‐C (the initial analysis team, all research staff). Each researcher became familiar with their assigned transcripts through a process of reading and re‐reading the data and making notes on initial points of interest. In phase two, the analysis team undertook line‐by‐line coding of their assigned transcripts using Microsoft Word. Coding was semantic and latent, meaning that the data was explored for surface‐level, participant‐driven meaning and implicit, researcher‐driven meaning. The four young researchers (AVS, HP, GC and LF) also coded a segment of a transcript. In phase three, the analysis team met to reflect on the coding process as a whole and to discuss the initial generation of themes. In phase four, NH‐S and CP‐C transferred the coded transcripts into NVivo, working collaboratively to develop the initial ideas into a set of more distinct and comprehensive themes and subthemes. In phase five, the analysis team met with the YRT to share and refine the generated themes in accordance with their own understandings. In phase six, MEL and NH‐S wrote up the final analysis, with feedback from the whole authorship team.

We worked within the epistemological and ontological framework of contextual critical realism, as we assumed we could comprehend and elucidate social phenomena whilst also acknowledging the influence of individual perspectives and experiences. Thus, we acknowledge that, at best, our understanding of the experiences of our participants is partial because social and contextual factors influence the sense we made of the findings. We posit that our findings reflect an underlying reality but are not necessarily objective or comprehensive and are influenced by the context in which they were derived (Tebes, 2005).

Our team includes clinical and research academics and YP. There is diversity in sex/gender, ethnicity and/or those living in deprived or isolated communities. Several team members have lived experience of mental health difficulties. Our common interests include adolescent mental health, with expertise around adolescent depression, help‐seeking, digital mental health interventions and qualitative methods. Some bring clinical backgrounds (MEL) and some work within an NHS research team (BT, TR, KJ and ECR). The young researchers (AVS, HP, GC and LF) all have an interest in psychology but no prior experience of working on research projects.

## RESULTS

### Description of the sample

We recruited 24 YP. Most participants were 17–18 years old and biologically female, with a range of gender identities, ethnicities and perceived social status (see Table 1). On the PHQ‐2, most participants (*n* = 17, 70.8%) scored ≥2, indicating at least moderate depression symptoms.

**TABLE 1:** Participant demographics characteristics.

Characteristic	Response options		*N*
Age (years)	13–14		0
	15–16		6
	17–18		18
Biological sex at birth	Female		17
	Male		5
	Prefer not to say		2
Gender identity	Woman/girl		16
	Man/boy		4
	Genderqueer		1
	Male‐to‐female transgender		1
	Agender		1
	Non‐binary		1
Ethnic group	White	9 English/Welsh/Scottish/Northern Irish/British 2 Other (Russian/Ukrainian; Jewish)	11
	Asian/Asian British	1 Bangladeshi 1 Indian 2 Other (Taiwanese; Afghan)	4
	Black/African/Caribbean/Black British	6 African 1 Other (no further information given)	7
	Mixed/Multiple	1 White and Black African 1 White and Black Caribbean	2
McArthur Scale of Social Status	Compared to families in the UK	Mean 5.17 (SD = 1.69), range 3–9	
	Compared to families in their school	Mean 3.92 (SD = 1.91), range 1–8	
PHQ‐2 score		Mean 2.29 (SD = 1.73), range 0–6	

*Note*: On the McArthur Scale of Social Status, 1 = top of the ladder, best off.

Abbreviations: *N*, Number of participants; PHQ‐2, Patient Health Questionnaire‐2.

### Analysis

**FIGURE 2:**
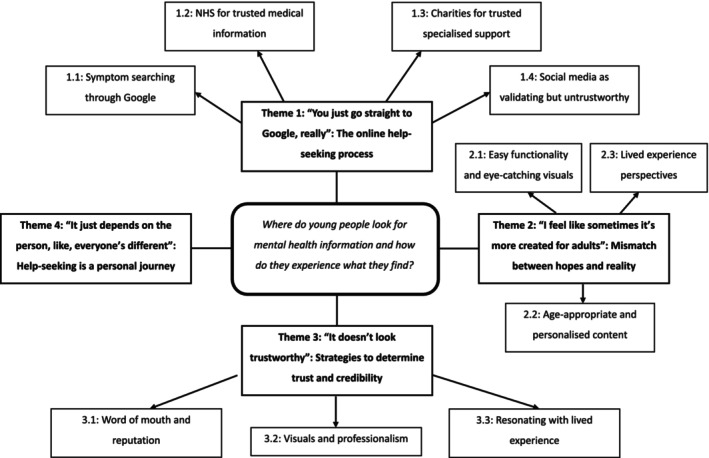
Thematic map.

Analysis generated four themes comprised of 10 subthemes that collectively explore YPs experiences and attitudes towards seeking help online: (1) The online help‐seeking process; (2) Mismatch between hopes and reality; (3) Strategies to determine trust and credibility; and (4) Help‐seeking is a personal journey. Each theme is described with illustrative quotes (see Figure 2 for a thematic map and supplementary materials S5 for additional illustrative quotes).

## THEME 1: ‘YOU JUST GO STRAIGHT TO GOOGLE, REALLY’: THE ONLINE HELP‐SEEKING PROCESS

YP described a variety of ways in which they looked for information online, ranging from medical websites to social media to forums. Collectively, most said that certain types of websites and content were sought for different purposes. This online help‐seeking process is explored through four subthemes.

### Symptom searching through Google

Almost unanimously, YP named Google as their first port of call when beginning to look for information online, implying familiarity and trust. Some explained that, as a well‐known search engine, they believed websites would require some kind of approval and validation to appear as a search result.

‘I think it is trustworthy and I think for something to be maybe approved and uploaded on this online searches, I will find it a trustworthy source’ (Sabi, 17–18).

Some YP described how they would only click on results from the first couple of pages of a Google search, equating their position with how many times a website had been accessed and therefore assumed trustworthiness. However, this was not the case for anything labelled as sponsored.

‘These are the [websites] that people go to, which is why it's on the front page’ (Luxseal, 17–18).

‘I don't tend to look at the sponsored stuff because I know they're exactly that—sponsored’ (Kiera, 17–18).

Symptom searching seemed to be a particularly common starting point, with solution‐focused or symptom‐based search terms (see Table 2).

‘I guess just doing a normal Google search, you know, what can I do if I have low mood, and seeing what comes up’ (Rose, 17–18).

**TABLE 2:** Summary of patterns for what YP searched for online, based on screen sharing.

Most participants initiated their searches on Google
Terms like ‘low’ and ‘mood’ were frequently used in initial search queries
Few participants searched for specific age or gender‐related information
Searches predominantly aimed at seeking practical advice and explanations for symptoms
Popular websites selected to click on included NHS pages and mental health charities like Young Minds, Samaritans and Mind

### NHS for trusted medical information

When Googling their symptoms, most YP expected to readily find information from the NHS; ‘normally NHS stuff comes up at the top.’ (Albertine, 15–16). Many explored this website first before accessing other search engine hits.

‘I start with the NHS website. I go on from there. It's like using it as Wikipedia’ (Gale, 17–18).

Access to truthful, factual information seemed to be an important part of YP's decision‐making process when choosing a website to visit. YP viewed the NHS website as trustworthy, due to it being an ‘*official*’ (Flower, 15–16) website associated with ‘*government stuff*’ (Kiki, 15–16), which meant that it would not ‘*directly lie about something.*’ (Gale). YP rated the NHS highly in this regard due to an awareness of how the NHS website is updated and maintained.

‘An NHS website is definitely the most trustworthy in my opinion, because I know it's like frequently monitored’ (Alex, 17–18).

However, whilst YP trusted the NHS, it was understood that the information available would only provide a medical overview and not go into any depth. For some, this surface‐level information was appropriate, particularly if it was their first attempt at help‐seeking; for others, potentially those with greater pre‐existing knowledge of mental health, alternative evidence‐based sources were preferred, like diagnostic manuals.

‘[The NHS] doesn't mention everything like if you go to the DSM‐5 for depression, it's a lot more stated in depth list of things, but it's‐it's a reasonable overview’ (Gale).

Some participants also criticised NHS websites for only providing information and not offering meaningful support, which was seen as particularly problematic for individuals with more severe difficulties.

‘It doesn't offer any real help […] it kind of just tells you the main symptoms and what…like, the really extreme cases, it doesn't really give you an option to help like right now’ (Sophie, 17–18)

### Charities for trusted specialised support

Charities were another source of information trusted by the majority of YP, often due to affiliations with other well‐known organisations, like the NHS and schools.

‘I'm doing university application stuff at the moment and when on the UCAS website, Mind is there and then I'm pretty sure I've seen Mind on the NHS website as well […] it's just a credible charity in terms of mental health stuff’ (Kiki).

Charity websites were perceived to provide more specialised support for YP, with more focus on specific mental health conditions like depression, or developed with a certain audience in mind, like children and YP. This somewhat tailored approach by charities was valued by YP, as the information provided felt more accessible and appropriate for what they were looking for.

‘It is Young Minds, so it is made for young people, whereas the NHS website isn't necessarily made just for young people’ (Aco, 17–18).

### Social media as validating but untrustworthy

Social media was a common topic across all interviews, with YP acknowledging that, due to its widespread use, it was often used as a platform for seeking mental health information. As a result, this is where they suggested the persona of Sally would hypothetically seek help from.

‘Everyone is using TikTok these days so [Sally] will probably seek help from TikTok’ (Fiona, 17–18).

However, unlike websites such as the NHS or Mind, social media was viewed as a double‐edged sword. On one hand, YP highlighted how useful social media could be for providing them with lived experience perspectives, which worked to reassure them, validate their own experiences and facilitate peer support.

‘Before I first sought support, I followed a lot of people who are in like on social media who are in similar positions as me, but who were working towards kind of recovery […] that kind of helped me to see that there was a future […] I feel like social media can be a negative tool, but also a pretty positive tool if used in the right circumstances’ (Maisie, 17–18).

Despite this, YP reflected on the many negatives of social media and ultimately perceived it to be untrustworthy. This was because, whilst validating, the individuals sharing their stories on social media were often not mental health professionals and were subsequently not trained to provide evidence‐based advice or support—but YP might not recognise this and assume that the information shared is accurate and trustworthy.

‘Sometimes it could do more harm than good, probably because, like even if you feel like it's someone that you relate to, it's not like they're a mental health professional’ (Nara, 17–18).

Underpinning some of these concerns was the perception that other YP, particularly younger adolescents, may not be aware of the potential unreliability of social media, or that the desire to be understood might override necessary caution.

‘I guess when you go on Instagram, you don't really think about the trustworthiness of it. Like if it‐if it conforms to what you think, then it's like reinforces that. Then you probably find it trustworthy’ (Zigzag, 15–16).

Not all participants agreed with this viewpoint, however, with some advocating for the savviness of YP and their understanding to ‘*[take] everything you see on social media, like, with a grain of salt*’ (Luxseal). Subsequently, whilst participants felt that the persona of Sally would use social media, she would be aware of its flaws.

‘So you said [Sally's] 15? So she'll probably look on TikTok as well. But she'll know that it's not the most reliable platform for stuff on mental health’ (Keira).

## THEME 2: ‘I FEEL LIKE SOMETIMES IT'S MORE CREATED FOR ADULTS’: MISMATCH BETWEEN HOPES AND REALITY

Participants spoke about their hopes and expectations for the information they would find online, and how this was often at odds with the reality of what they found. Three subthemes were generated to highlight different types of mismatch.

### Easy functionality and eye‐catching visuals

Most YP wanted to access websites that were visually appealing, intuitive to navigate and clearly structured. Formatting choices, such as chunking information into bullet points and highlighting key phrases, were perceived to enable easier access, whereas use of bright colours and multimedia helped to maintain engagement.

‘It's very colourful, it's very highlighted, it's got titles, it's got like sort of separate sections at the bottom there it's got little hearts, which makes it just makes it feel a little bit more homey than the NHS […] it feels a bit more understandable, a bit more readable’ (Matilda, 15–16).

Unfortunately, this kind of functionality was not present in some of the websites YP typically visited, such as NHS websites. Long paragraphs, information presented on a singular page, and formatting choices like *‘big white background, big black text’* (Gale) were experienced as overwhelming and intimidating, and prompted disengagement from the help‐seeking process.

‘That first block of text is just a bit difficult to read and I think as a result, even if it that has helpful information on it, people are more likely to scroll through and try and find something a bit shorter’ (Taylor, 17–18).

Some YPs emphasised the importance of keeping the target audience in mind when creating content. Upon reflection, they doubted how accessible some of the information would be if they were experiencing symptoms of depression, like the persona of Sally.

‘Everything is so well explained in small points, I think it would be easy to read through, especially when you're in a bad mood. When you're in a bad mood, [you] wouldn't have fun reading through a lot of words’ (Fiona).

### Age‐appropriate, personalised content

YP hoped to find content that was appropriate for their age group. Some participants discussed this in the context of how information was written, preferring simple language that was not overly medicalised or too formal. Participants highlighted that ‘*information for young people needs to kind of be as digestible as possible*’ (Maisie).

However, ‘simple’ did not mean that they wanted to be patronised. YP emphasised that ‘*different age ranges would want different information presented differently*’ (Matilda) and information that may be appropriate for a younger teenager may not be well‐received by an older teenager.

‘Say you're 12 years old, [the CAMHS website] might seem relevant but I feel like when you get more from like 16 to 18, just it almost seems like with the wording they've got, it's almost pampering you […] they're trying to cushion it too much’ (Jago, 17–18).

Personalisation seemed to be important: many YP wanted the website they visited to feel like it was written with someone like them in mind. Whilst they recognised it would be difficult for websites to tailor content to their individual needs, YP highlighted small details that could make a significant impact, like pronoun use and images of YP.

‘It feels more personal because it's got like, pronouns such as ‘you’. So it's more—it's more relatable’ (Toyosi, 17–18).

In contrast, some YP found websites that contained generic information impersonal and frustrating, particularly if it included advice that they had heard before and found unhelpful.

‘I was disappointed when it came to the tips. I don't know what I was expecting, but it just seemed like [doing] activity every day. I've heard that a million times’ (John, 17–18).

A solution some participants suggested was to have co‐created content. YP's involvement boosted participants' trust in a website because it was perceived that the content would be more relevant and applicable.

‘I think sometimes if it's just psychologists creating the guides, it could be hard to have a true understanding of‐of what it's like to be young person today […] with as much research as you can do in everything you can't actually be in a young person's brain’ (Maisie).

### Lived experience perspectives

Many YP highlighted the value of lived experience perspectives and were enthusiastic when these were available on websites in formats such as blogs and videos. These personal stories prompted some participants to reflect on their own experiences of seeking help online and their desire to find someone who they could relate to.

‘There's like a story as well. Like I've seen these quite a few times just to like see that you're not alone and you can get help for it […] in my case it did help me feel like I was less alone and there was like a light at the end of the tunnel’ (Alex).

Lived experience perspectives were understood to provide YP with hope and a sense of reassurance. When this reassurance was absent, participants highlighted how easily YP could begin to doubt themselves and fear abnormality.

‘Reading through this, I remember when I was first struggling with things and I was looking online for information. I was like, oh my God, something's wrong with me […] I've got depression and I was like, hold on, how many people have it? Is it abnormal? […] it's nice to have something that's like, it's not all that bad, you know, it's like a lot of people struggle with it, there are things to help’ (Matilda).

## THEME 3: ‘IT DOESN'T LOOK TRUSTWORTHY’: STRATEGIES TO DETERMINE TRUST AND CREDIBILITY

YP employed various strategies to determine the credibility of the websites they visited, which in turn influenced their perceptions of trustworthiness. These strategies are explored through three subthemes.

### Word of mouth and reputation

As highlighted in theme 1, many YP implicitly trusted websites associated with well‐known organisations, including the NHS and other healthcare organisations, government websites, schools and universities. Participants indicated that they were likely to trust information if it was developed, shared or recommended by these sources, suggesting the importance of reputation.

‘When I was at school we had assemblies from like Kooth and ChildLine and that sort of stuff, so like going somewhere like that could be like a good resource’ (Luxseal).

‘It leads to a .org website. I've not ever personally myself heard of [lab], but.org websites are usually trustworthy’ (Gale).

Word of mouth also played a role, with some YP describing the influence of recommendations from those around them. Friends particularly seemed to hold a lot of sway, alongside authority figures like teachers and healthcare professionals.

‘Definitely when I'm looking for support and I'm online, I will ask my friends and see what they recommend and see what they don't recommend’ (Aco).

‘I feel like if it's like an official website or app, well, it's been recommended by a teacher or trusted adult, then you'll be more likely to believe it’ (Sophie).

Furthermore, an unfavourable reputation discouraged use of particular resources.

‘Obviously CAMHS hasn't exactly got the best reputation [so] it's not a website young people would go to […] CAMHS says they're going to do a lot of things and then don't do it, and I know a lot of people who um have just had really bad experiences with CAMHS’ (Kiera).

### Visuals and professionalism

Whilst reputation was important, visuals also had a significant impact. When asked about perceived trustworthiness, many participants talked about the look of a website, such as the use of colour, fonts and the user interface. If a website seemed to lack professionalism or looked like something a YP could have created themselves, they were less likely to trust it.

‘It doesn't look as well made as like a website or other sort of flyers I've seen […] I think it makes it seem a bit less trustworthy’ (Beyonce, 17–18).

The presence of a logo could help to mitigate a lack of professionalism in some instances but not others, linking back to the importance of reputation combined with a carefully designed website.

‘I mean it's got NHS on it, so yeah, trustworthy’ (Rose).

‘I mean it says that it's a university, so I would think it's somewhat trustworthy, but it doesn't look trustworthy like it looks a bit like it's been cobbled together by maybe someone in secondary school, like a poster that they'll put up in their form room. It doesn't seem the most professional looking’ (Aco).

### Resonating with lived experience

Beyond offering reassurance and inspiring hope (theme 2), YP valued lived experience perspectives because they also functioned as a strategy for determining trust. Several YP described how reading or watching something they could relate to increase the perceived authenticity of a website.

‘I guess like if it feels like relevant to her, like, if there was a quote or something‐someone like her was going through something similar and they were like, ‘all [these] pages and information really helps me’ then she might like find that more reliable’ (Maeve, 17–18).

The reputation of the person sharing their lived experience also impacted perceived trustworthiness, with some YP linking this back to discussions of social media and the role of influencers (i.e. people like them or people followed by many others).

‘With TikTok, you're not gonna believe everything someone says to you. So if they have maybe a bigger following, then people are more likely to believe them, but you still gonna take it with a pinch of salt’ (Sophie).

## THEME 4: ‘IT JUST DEPENDS ON THE PERSON, LIKE, EVERYONE'S DIFFERENT’: HELP‐SEEKING IS A PERSONAL JOURNEY

Underlying participants' experiences and perspectives of looking for mental health information online was the understanding that help‐seeking is an individual process that differs on a case‐by‐case basis. As such, no single resource would be appropriate for all YP.

‘I don't know if any of them could be a 10 out of 10 just for the pure sense that obviously mental illness is very unique to every single person and nothing's ever going to work for everyone’ (Aco).

YP discussed how the needs of one person might vary considerably from the needs of someone else and that this depended on the broader context of their help‐seeking. For example, YP highlighted that information sought by someone new to help‐seeking might differ from information sought by someone who had reached out for support before. This could influence how appropriate and helpful the information was perceived to be.

‘I think if someone new to look[ing] into mental health came, they'd be very overwhelmed. But for someone who knows what they're looking for who is trying to find something, it's a pretty good source of information’ (Gale).

Individual differences could also stem from the type of information wanted. For example, some YP might want in‐the‐moment support, whereas others might be looking for signposting.

‘It just depends if the person wants to learn or if they're coming specifically for like coping mechanisms or they have something in mind that they wanna gain from the resources’ (Sophie).

YP suggested that depression severity could also influence this process. Someone with milder symptoms might be in a position where self‐help resources are perceived as beneficial and a good first step, whereas someone with more moderate‐to‐severe depression might struggle to find the motivation or energy to engage with information.

‘There's a lot of like self‐help resources, so if [Sally] feels like it's something she can temporarily get under control or she doesn't feel exactly ready yet to go and talk to someone’ (Alex).

‘Because [Sally is] experiencing low mood, some of it seems like it's too much […] like doing sport, she stopped dancing, it seems very not appealing because she's just too tired to do it, but talking to people I think she would consider’ (Aco).

Participants highlighted that these individual differences might mean that YP search through multiple resources before finding something that worked for them. In this way, online help‐seeking could be seen as a trial‐and‐error initiative.

‘For a lot of people, they will exhaust a few options online, so they'll go through all of the different websites and maybe then they might not find it helpful to them because obviously each person to their own, it really differs on how each person is feeling, so it may be a case of some information may not appeal to them, and some might’ (Matilda).

## DISCUSSION

We found that when YP look for mental health information online, the help‐seeking process tends to start with symptom searching on Google, with NHS websites perceived as useful for surface‐level trusted medical information and charities as sources of more in‐depth information, tailored to specific user groups. We also found that social media was often a source of information and support, valued for the validation it could provide, although often perceived to be untrustworthy. By exploring what our participants thought of the information they found online, we noticed that what YP hoped to find and the reality of what they found were often unaligned. They wanted eye‐catching visuals, easy website functionality, age‐appropriate and personalised content, the inclusion of lived experience perspectives and some immediate solutions to try. They used a variety of strategies to determine the trustworthiness and credibility of online information, including the reputation of a source, the look and feel of the information and cues that indicated a fit with them as YP. However, YP emphasised that individual differences could affect the help‐seeking process and ultimately concluded that no single resource would work for everyone.

YP in our study distinguished between different sources of information as serving different purposes. The different functions of online information and support (Pretorius et al., 2019) seemed to be related to the type of information provided; for example, symptom information was best obtained through the NHS websites our participants visited, whilst more detailed and tailored information was fulfilled by charities and social media, especially for lived experience accounts and/or a sense of connection with others. Previous qualitative studies have highlighted the value of social media for peer‐led learning (Berry et al., 2024) and of lived experience accounts as not only potentially motivating and empowering but also demoralising and overwhelming (Winstone et al., 2023). There is an interesting contradiction in our participant narratives: on one hand, YP talked about the benefits of lived experience and felt frustrated by the lack of such content; and yet, on the other hand, they also sought signs of authority and professionalism. This contradiction may be due to a degree of social desirability (Bergen & Labonté, 2020), expecting that we as researchers may expect certain answers and wanting to fit their responses to this for social approval. Alternatively, it may be a genuine cognitive dissonance (Cooper, 2012), with YP feeling drawn to both types of creators of content, even though these may be at odds with each other. Mixed views have also been found in other studies looking at online support for suicide prevention, for example with participants indicating that formal sites do not necessarily meet the different needs of users, especially when in crisis, and that lived experience accounts can be both motivational and inspirational and also challenging and disheartening (Cohen et al., 2022; Winstone et al., 2023), for example. This suggests that there needs to be a balance between different types of information and that different sources may be helpful depending on the individual and their state in the moment when they are looking online.

A commonality amongst our participants was that the look and feel of online resources was important. As posited by feelings‐as‐information theory (Schwarz, 2012), feelings are used as a source of information themselves within information processing, whereby different feelings provide different types of information and influence the conclusions we draw. Most of the resources our participants found, as well as those we showed them, did not fully match their expectations or ideals for what they hoped to find. In particular, NHS websites tended to be experienced as trusted but inaccessible due to the lack of visual appeal, limited functionality for navigating through the content and the use of complex language. This is similar to findings from other studies in YP (Cohen et al., 2022) and adults (Biddle et al., 2020) in distress. This highlights the value of user design, with particular attention to aesthetics, functionality (Maddison et al., 2023) and the use of appropriately plain language without excessive jargon (i.e. scientific language). Jargon use has been found to negatively affect engagement with science information generally (Dews et al., 2024), specifically affecting processing fluency (even if definitions are included) and self‐identification with the information in adults (Shulman et al., 2020). Furthermore, plain language is preferred by YP (Stallwood et al., 2023). Given that NHS England has guides on designing for inclusion (NHSEngland, 2021) and for creating accessible websites in primary care (NHSEngland, 2022), it is surprising that NHS provided information did not seem to better meet our participants' needs, indicating that codesign and user experience work is needed.

Extending beyond the existing guidance, we identified several ways in which those developing and sharing information for YP could specifically meet their needs and preferences. They want practical advice that offers immediate help and explanations that are understandable and relevant, with information presented in a way that is visually appealing and easy to navigate. Again, this is consistent with previous studies (Biddle et al., 2020; Cohen et al., 2022). YP want information from experts but also from peers who are seen as more relatable (YSkills, 2023) and inspiring, which instils hope. This aligns with self‐determination theory, which posits that individuals are driven by their innate needs for autonomy, competence and relatedness, which influence their motivation and well‐being (Deci & Ryan, 2012).

We found that YP were shrewd and knowledgeable about judging online information. Almost all used Google to search for information, similar to other research that found that Google is familiar and easy to use (Best et al., 2016). They also showed a good understanding of how Google works as a search engine—for example, they avoided sponsored pages and understood that websites higher up in the search results had been more frequently accessed. When judging websites, some YP looked out for suspicious URLs before deciding whether to click on a website. They also made good use of visual indicators (e.g. professional appearance of website, recognisable logos) and ‘official’ status (i.e. from a reputable and known organisation) as indicators of quality, which were cues to inform their judgements of trustworthiness. This is consistent with other studies of YP (Best et al., 2016) and adults (Fisher et al., 2023) and is in line with the concept of source credibility (Pornpitakpan, 2004) and the institutional trust aspect of the comprehensive model of information seeking (Liu & Yang, 2023).

Furthermore, despite the predominance of literature expressing concern about the risks YP are exposed to in online spaces and the potential detrimental effects on their mental health (Keles et al., 2020; Savoia et al., 2021), our participants saw the positives of social media and found lived experience accounts as easy to relate to and validate. However, they were also aware of limitations, such as influencers not being trained professionals. Similar to a recent study of YP with Type 1 diabetes (Berry et al., 2024), our participants talked about the problem of algorithms, which means that users see more of similar content rather than a balanced perspective. Again, this may be explained in part by social desirability and by the hypothetical nature of the scenario; some of our data hinted that reality may be different and that in the moment when looking for support, YP may not consider trustworthiness, and their desire for connection may override caution.

## STRENGTHS AND LIMITATIONS

Initially, our study advert mentioned depression, even though it was not an eligibility criterion. We subsequently removed this explicit reference to depression from study adverts after feedback from our YPAG that it may deter YP who did not self‐identify as having depression. This means that some of our participants may have been in the mindset of someone looking at a scenario from a position of wellness, which may be quite different from the mindset of someone with a low mood reaching out for help and connection. Although we did not ask our participants whether they had current mental health diagnoses, we did use a brief depression scale (PHQ‐2) to describe our sample, who ranged from scoring the minimum possible to the maximum possible on this scale. We also only recruited YP who could take part in a virtual interview and therefore may not have incorporated the perspectives of those who could not do this.

Although we had intended to use some purposive sampling to ensure sample diversity (e.g. gender identity, ethnicity and deprivation), we contacted all those who opted in during the recruitment period. Whilst the resulting sample was diverse in many ways, we did not recruit any 13‐ or 14‐year‐olds. As posited by the ladder of online participation, older teens tend to be increasingly active online (Livingstone et al., 2019); therefore, it may be important to explore the ways in which younger YP use the Internet to look for mental health help in future studies. In addition, the participant group was primarily female. As such, there may be differences in the preferences and perceptions of different sex and gender identities relating to mental health information, which we were not able to explore in this participant group. Further work in this area, however, may provide valuable insights into how we can better support young male mental health and reduce sex and gender inequalities in mental health outcomes (Sagar‐Ouriaghli et al., 2023).

Like other online studies offering incentives for taking part (Pellicano et al., 2023; Ridge et al., 2023; Wang et al., 2023), we encountered many fraudulent responses at the sign‐up stage, both from bots and individuals who were not who they said they were. This meant that we had to introduce additional validation checks, including insisting that participants turned their cameras on for at least some of the interview. We acknowledge that this could have been off‐putting to some YP, including those who may be most likely to seek help online in anonymous spaces.

Whilst we assume that having a young researcher in the interviews may have helped to set the participants at ease, including by framing their questions in familiar language and sharing their knowledge about navigating sites and processes during the interviews, it did mean that there were two interviewers involved, which may have been intimidating, and we did not specifically ask participants about their experiences of this although most participants informally stated that they found the interviews to be an enjoyable experience.

## IMPLICATIONS

To increase the accessibility of help and decrease stigma, many UK CAMHS have adopted the THRIVE framework (Wolpert et al., 2019) as a working model for delivering care. THRIVE's first tier is ‘getting advice and signposting’ for mild to moderate difficulties (i.e. ‘early help’). This includes promoting resilience through self‐management and one‐off contact. Our findings highlight that online information and support may be a key part of this first tier. We have coproduced guidelines based on our findings and the experiences of our YRT (see Table 3) to assist in developing online information and support for YP. However, we also emphasise the importance of coproducing information with YP to ensure that it is engaging, relevant and credible to the target end users, particularly as the needs of specific groups such as LGBTQIA+ or ethnic minorities may be different. Further research is needed to understand the information needs of specific groups, particularly those who are more vulnerable to experiencing mental health struggles and/or less likely to access other sources of information and support. To make information easily findable, those developing and sharing information online should bear in mind that YP do not necessarily start by knowing diagnostic labels such as depression, and so the terms they search for and what they choose to look at may fit more with how they see themselves. Recent work in the United Kingdom that found YP with elevated depression symptoms described themselves as ‘tired’ (20%) and ‘sad’ (14%) but also ‘kind’ (11%) and ‘funny’ (16%) (Hards et al., 2023); such self‐evaluation studies could be informative when developing resources.

**TABLE 3:** Coproduced guidelines for creating and sharing mental health information for young people online.

Where to share?	On the social media platforms which YP are currently using (e.g. Instagram, TikTok and YouTube) In the places YP already look (e.g. NHS websites and charities) Through organisations that YP trust and regularly come into contact with (e.g. schools, mental health services) Ensure websites are findable on Google, especially if YP search for symptoms like'low mood'/'sad'/'tired'
What to share?	Validation and normalisation of how they are feeling, including lived experience accounts Reasons for why they may be feeling a certain way (e.g., possible triggers) Words/phrases and clear definitions that could help YP to talk to others about how they are feeling Sources of support, including in person and online, and those that are available 24/7 Give different options and ideas of things that may help Simple small steps that YP can take to feel better (but avoid difficult or overwhelming tasks) Clearly signposted information aimed at friends and family
How to share? (look and feel)	Use plain language that is easy to understand but not patronising Make it colourful, but not overwhelming Write in smaller sections rather than huge chunks and use bullet points Include links to jump to specific sections rather than scrolling Include pictures of YP and lived experience accounts (case studies) from YP Say who produced the information, include logos and links to institutions Ensure that it looks professional Check readability of the website and accessibility for all Use multimedia to share information in different formats (e.g., videos, podcasts)

Abbreviation: YP, young people.

However, whilst a range of online information and support should be available, for some YP, being signposted to online help can be experienced as dismissive and subsequently invalidating (Maddison et al., 2023). Additionally, YP with mental health difficulties have also described how some information can be upsetting, triggering and can compound the problem (YSkills, 2023). Therefore, online information should offer guidance about how to decide if they need to seek further help and where to do so. Barriers to access in our work and in other studies with young adults (Biddle et al., 2006, 2007) have found that YP struggle to decide whether their depression symptoms fall within the medical remit, and therefore may not think that primary care (e.g. GPs) is an appropriate source to consult (Biddle et al., 2006). YP also seems to struggle with knowing how to define their symptoms and how to decide if they are bad enough to be ‘real’ and in need of treatment. Online resources may be used to assist with this important stage of lay diagnosis as a stepping stone to disclosing and seeking help from others offline. However, we need to be cautious about the potential of overdiagnosis of normal emotions as per the prevalence inflation hypothesis (Foulkes & Andrews, 2023).

## CONCLUSION

Our study highlights the diverse avenues YP utilises for seeking mental health information online, including Google searches, NHS websites, charities and social media. However, discrepancies between their expectations and actual findings often lead to dissatisfaction. To address this, future efforts should focus on enhancing the credibility and user experience of online resources. This includes ensuring visually appealing and user‐friendly interfaces, providing age‐appropriate and personalised information, and incorporating insights from peers with lived experience. Moreover, codesign and coproduction approaches are essential for developing online platforms that effectively meet the needs of YP and foster their engagement with mental health support. Moving forward, research should continue to explore innovative strategies for improving the accessibility, reliability and relevance of online mental health information and support for YP. We also need to understand more regarding the needs of specific, underserved populations.

## AUTHOR CONTRIBUTIONS

**M. E. Loades:** Conceptualization; investigation; funding acquisition; writing – original draft; writing – review and editing; methodology; project administration; formal analysis; data curation; supervision; resources. **N. Higson‐Sweeney:** Methodology; formal analysis; writing – original draft; writing – review and editing; data curation. **B. Teague:** Conceptualization; investigation; writing – review and editing; formal analysis; supervision; project administration. **J. Leas:** Investigation; formal analysis; project administration; data curation. **C. Payne‐Cook:** Writing – review and editing; data curation; formal analysis. **A. V. Slastikova:** Formal analysis; investigation; writing – review and editing. **H. Peel:** Formal analysis; writing – review and editing; investigation. **G. Chamberlain:** Investigation; writing – review and editing; formal analysis. **L. Ferguson:** Formal analysis; writing – review and editing; investigation. **K. Janes:** Investigation; writing – review and editing. **T. Rhodes:** Investigation; writing – review and editing. **E. C. Roupa:** Investigation; writing – review and editing. **L. Biddle:** Investigation; writing – review and editing.

## FUNDING INFORMATION

MEL (Advanced Fellowship 302929) is funded by the National Institute for Health Research (NIHR) for this research project. The views expressed in this publication are those of the author(s) and not necessarily those of the NIHR, NHS or the UK Department of Health and Social Care.

## CONFLICT OF INTEREST STATEMENT

No conflicts of interest to declare.

## Supporting information


Data S1:

## Data Availability

Data collected during this study may be made available to other researchers for future data analyses following publication of primary findings at the conclusion of the study. We will share data with other investigators within and outside our university upon submission of a reasonable written request.
