# Supplementary material for: What do they look for and what do they find? A coproduced qualitative study on young people's experiences of searching for mental health information online
Source: Psychol Psychother. 2024 Oct 14;98(2):373–95. doi: 10.1111/papt.12550 (PMC12065074; doi:10.1111/papt.12550)
Supplement: Supplementary file 1 — Data S1: [file PAPT-98-373-s001.docx]

**Supplementary Materials**

**S1. Example adverts shared on social media** (Instagram including a paid boost post for 1 week during August 2023, targeted at age 13-18s in the UK; Twitter/X; LinkedIn; Facebook). Adverts were predominantly shared by social media accounts specifically created for this study, and also by the research team’s personal accounts.
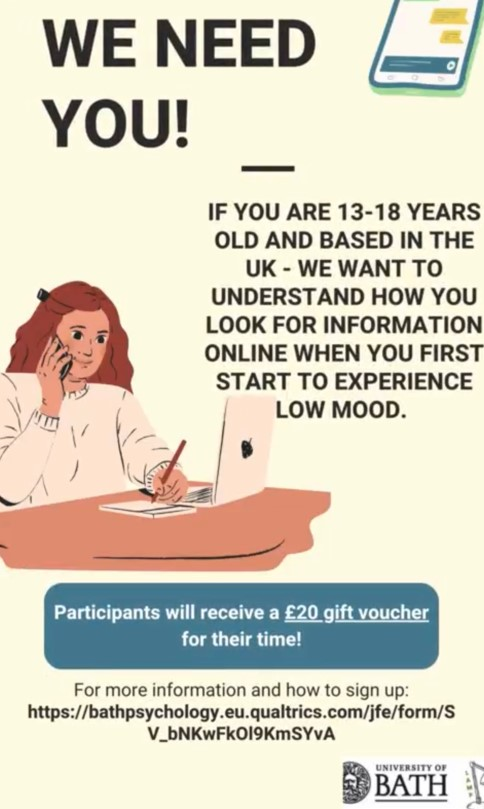


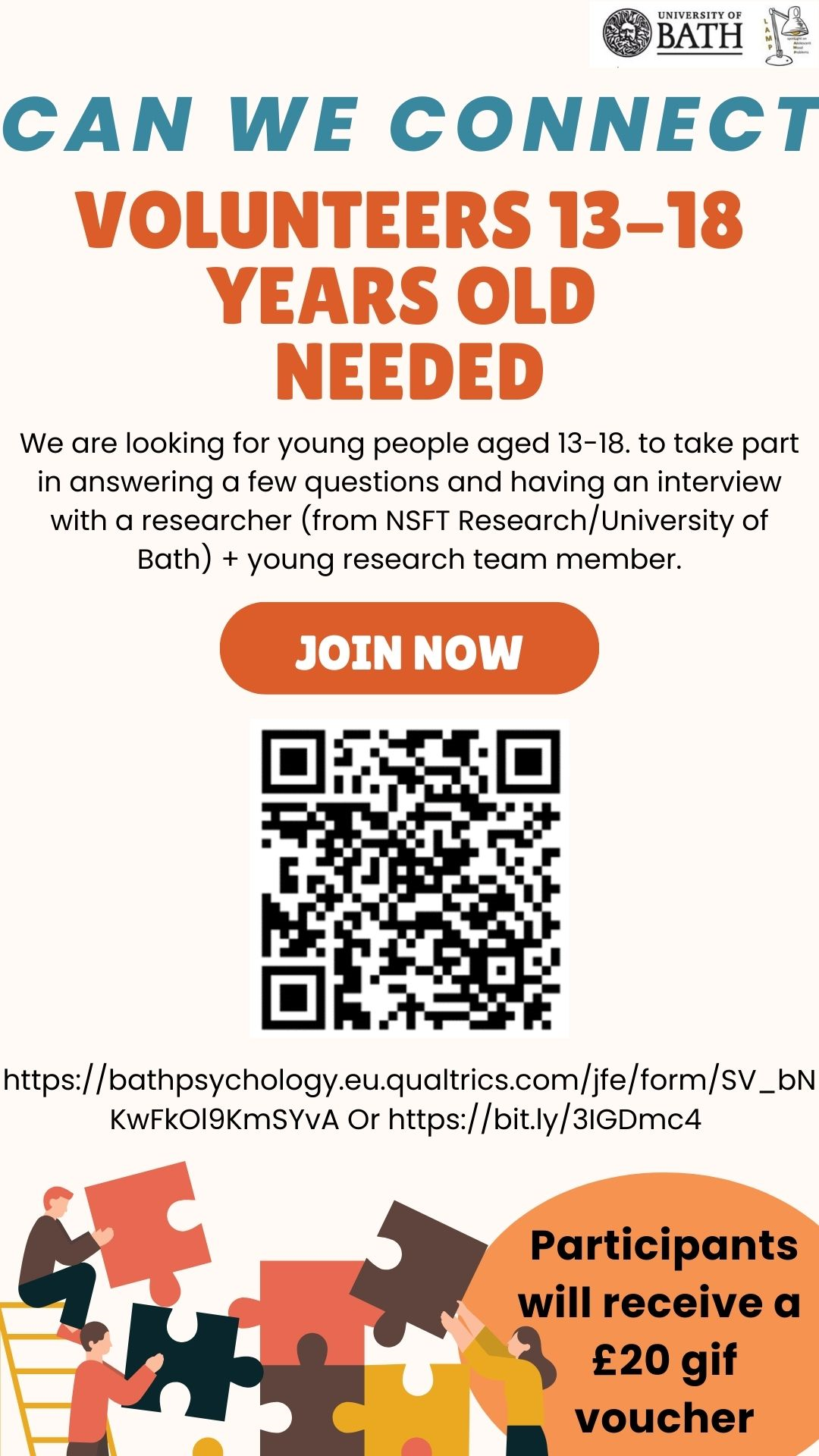


**S2. Details of Bot checks processes implemented**

Standard Operating Procedure for checking the legitimacy of sign ups via Qualtrics CWC project 1

# Problem:

- Many sign ups on the Qualtrics CWC project 1 which captures expressions of interest and takes consent appear not to be legitimate/are questionable
  - Some are bots – incomplete and can be spotted due to lack of completion of required information e.g. email address, creating a pseudonym
  - Some are real people but not young people in the UK (i.e. do not meet eligibility criteria)

# Process for dealing with this:

- Step 1: Qualtrics
  - Built in RECAPTCHA score - if this is less than 0.5 it indicates that the respondent is illegitimate.
  - Index prevention - this just means that the survey wouldn’t come up on search engines if the terms were input into one.
  - Also used the manual RECAPTCHA question - if respondents failed this, then they cannot move on past it, although it’s not forced response, so they don’t have to answer it anyway (it does not allow you to force response it).
  - Using the Qualtrics function for response quality:
    - There are duplicate responses – filter these out.
    - Then there are people who Qualtrics has flagged as potential bots – we ignore these too and assume they are bots.
  - People who we are sure about e.g., people who have left phone numbers or who have left an email and created a convincing pseudonym – proceed to contact to arrange an interview
  - People who we are unsure about
    - Contact by email as follows:
      - Many thanks for your interest in taking part in the Can We Connect Study. This is open to 13- to 18-year-olds who are living in the UK. Please can you confirm that this applies to you? Note that we will ask you to turn your camera on during the interview and will not be able to send you a thank you voucher if you cannot do this/prove to us that you are who you say you are.
- Step 2: check responses to the emails
  - If they email back, we need to judge whether they are genuine participants.
    - E.g. if you see a batch of responses all using exactly the same wording, then file in the CWC not genuine subfolder and ignore.
    - For those that seem genuine, please email them back to say thanks for confirming and proceed to arrange interview.
    - If unsure, email further questions to them e.g. where in the UK are they? -> return to start step 2 again – continue until a decision is made.

Tips:

1. If you ask people where they are based, if they are very vague about it, then it tends to be a Bot. So they may answer, but say something like “the north of the country” or “near London” or something like that and won’t reveal anything further – some language that isn’t typical basically!
2. Be careful of emails which are in the format of firstname/lastname + a couple of numbers @gmail.com (i.e. [alvingrim68@gmail.com](mailto:alvingrim68@gmail.com)) – although some of these are real, it usually signals a mass-created bot email address with an impersonator at the end of the line.
3. Also, there is a tendency with international operations running Bot/impersonator accounts to use unusually retro names that aren’t in common use nowadays in young people. Again, these names may be real but otherwise can be a sign of a bot operation.
4. Also look at the timings that the signups are to Qualtrics – if there is a flurry of sign-ups over a couple of hours or days, it usually signals a spam operation.

**S3. Response options given for gender identity question**


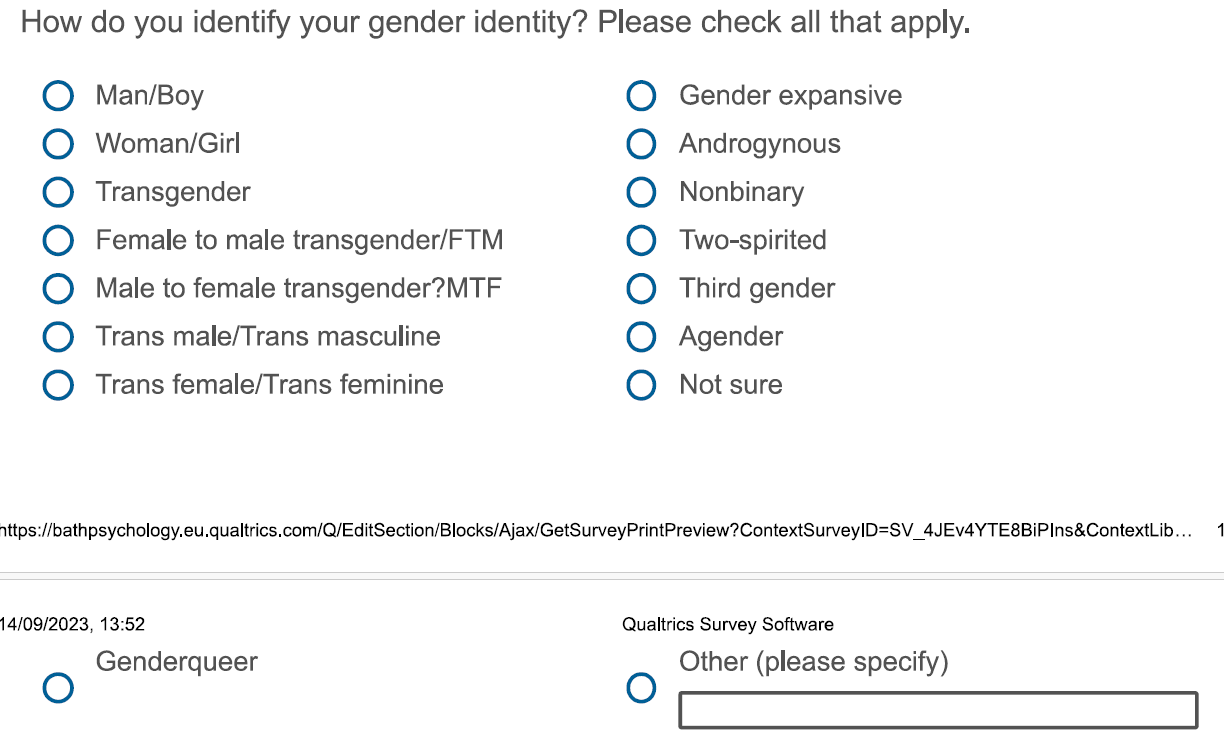


**S4. Can we connect study topic guide sections relevant to current analysis**

*note this can be used flexibly* - the interviews should be a conversation between the interviewers and the young person participant.

# Interview Part 1: Persona exercise

- *Young Research Team interviewer to lead, research team member to take observational notes and support conversation/add probes as necessary*
- Participants will be presented with a ‘persona’, generated by the young research team. This persona describes a young person who is beginning to struggle with low mood and not enjoying things they used to enjoy anymore. Instructions given to young researchers in generating personas:

A persona is fictional character.

Here is an example persona I previously generated with PPI input - Sally is a 15-year-old girl from a mixed race background. Over the past few months, Sally’s parents and teachers have become increasingly concerned about her low mood and withdrawal from life; she has been less interested than usual in seeing her friends and is reluctant to go out and about with family members, preferring to stay at home. Her form tutor has noticed that she is much more tearful than usual at school, and even relatively minor things, like getting a B grade in a test rather than an A, have been very upsetting to her; she also told her mother that she doesn’t think she is good enough. She continues to go to school, but isn’t doing as well as she usually does, and her grades have slipped. She has opted to give up dancing, which she previously enjoyed.

The key features we need the persona to display are:

- starting to struggle with low mood, not enjoying things so much any more. Finding it hard to sleep.

- doesn't feel ready to talk to anyone yet

- Suggest keep it simple, don't add too many details that aren't needed.

- Probes:
  - Where do you think they [persona] first look for help?
  - Where would they look for help online?
    - Probe for:
      - how they search online and on social media
        - where?
        - How? Which platforms? What would they be looking for on each platform/what would different platforms offer?
      - what influences their perceptions of the information they find.
      - Is this similar to where you would look/have looked in the past?
      - Where else might you look/what else would you do if it were you who was having difficulties a bit like this?

# Interview Part 2: Searching for help online

- *Research team member to lead*

Next, we would like you to open your internet browser and share your screen (give instructions if needed).

Please show us how you would search for help if you were struggling with your mood.

(note what search terms they use, what search engine)

- What do you think of what the search has turned up?
  - Now, we please open 3-4 pages that have come up in the search, the ones you would be most likely to choose to open if you were looking for information yourself
    - what do you think of this information?
    - probe for relevance (how well does it fit what you are looking for), trustworthiness (how much do you trust the information), and credibility (how accurate and believable is the information?).
      - For each page they open, use 0-10 ratings for these dimensions and ask them why they have rated it at this

Thanks! You can stop your screenshare now.

# Interview Part 3: Thoughts about public health information

- *Young Research Team interviewer to lead, research team member to take observational notes and support conversation/add probes as necessary*

Now, we would like to show you some information. [Research team member interviewer to screenshare]

1. NHS information <https://hampshirecamhs.nhs.uk/issue/depression-professionals/>)

- What do you think about this information?
- How trustworthy is it?
- How relevant is it for a young person?
- How credible/believable is it?
- What do you like about it?
- What don’t you like?
- How could this website be improved for young people?

1. Charity information <https://www.youngminds.org.uk/young-person/mental-health-conditions/depression/>

- What do you think about this information?
- How trustworthy is it?
- How relevant is it for a young person?
- How credible/believable is it?
- What do you like about it?
- What don’t you like?
- How could this website be improved for young people?
- What could be good about this kind of offer?
- What kinds of topics might be useful?

[NOTE participants were then asked to think aloud in response to being shown a study advert for a single session intervention study, and to share their thoughts about online single session interventions with reference to the persona presented at the beginning, this has not been included herewith as the data arising from these sections of the interview were not relevant to the current paper]

S5. **Table S5.** *Additional illustrative participant quotes by theme*

| Theme | Participant quotes |
| --- | --- |
| ***Theme 1: The online help-seeking process*** | |
| - 1. Symptom searching through Google | “So I’d use Google and [I’d] just type in something there and look for a solution and I'm-I’d probably use any website that pops up.” (Fiona)  “It depends because [Sally] could either get resources from school that are online that they forward her to, or she could just go on Google and look at whatever comes up.” (Sophie)  “[Sally] might just start like type ‘why am I uh, depressed’ or ‘why do I have low mood’ and then just pick the first few websites.” (Toyosi) |
| 1.2 NHS for trusted medical information | “I feel like for me personally, I would-I would probably check the NHS website first before I start, like, delving off into other places.” (Toyosi)  “If I see something NHS, I immediately trust it pretty, pretty strongly, and I know it's all kind of backed up with [an] evidence base.” (Maisie)  “I think it’s from NHS and I don't think er they can put the wrong information.” (Lan) |
| 1.3 Charities for trusted specialised support | “[The] NHS [website] was more sort of succinct and like very clear, especially with like the checklists, and [the Mind website] [...] feels like it’s more information which someone might want.” (Rose)  “I am aware of Young Minds, like they've come to my school before, so I do know that [they are] quite a reliable charity and, yeah, I think that they're quite reliable.” (Keira) |
| 1.4 Social media as validating but untrustworthy | “If she didn't know any better, [Sally] would probably go to social media because that is purely what young people these days mostly know. They mostly understand social media.” (Matilda)  “I don't think it's right for her to base her evidence or base her conclusion on what she gets from the social media.” (Ami)  “It's not always the most trustworthy, and I think it's difficult to-if you're following lots of accounts surrounding mental health to make sure that it's kind of a positive and not a triggering [...] seeing someone in intense distress might not be the most helpful.” (Maisie) |
| ***Theme 2: Mismatch between hopes and reality*** | |
| 2.1 Easy functionality and eye-catching visuals | “I like that there's a lot of pictures that kind of keeps me like figuring out what's-what's going on and it's not just words.” (Kiki)  “I can see there's like videos [there] as well and it gives you like podcasts and apps. So I think it's good that there's different options because not everyone processes like information in the same way.” (Nara) |
| 2.2 Age-appropriate, personalised content | “I like that it has the picture of the teens, that makes it feel like there's other people who I could relate to a bit more because [...] they look about my age range.” (Matilda)  “I can tell [Young Minds is] quite more like catered towards young people than anyone else, just because I get more information and I can know exactly what I'm signing up for.” (Alex) |
| 2.3 Lived experience perspectives | “And there's some more quotes here, and there's like a story as well. Like I've seen these quite a few times just to like see that you're not alone and you can get help for it [...] like they have little videos where they have like a group of people talking about a particular condition, and these can also be quite helpful because there's quite - anecdotal points of view like they're sharing their own views and how they cope and get help for it.” (Alex)  “This is just personal preference, but I think when you go onto a website like this, I would prefer to have a video at the beginning or like a link to a video. Just so someone can give you a very general explanation like not too long like a two and a half [minute] video, three minutes, just going-just trying to motivate you and [show] you’re not alone, other people have gone through the same thing.” (Jago) |
| ***Theme 3: Strategies to determine trust and credibility*** | |
| 3.1 Word of mouth and reputation | “It's from a clinic, it's more trustworthy to me.” (Beyonce)  “I think friends tend to influence a lot of decisions that people make [...] they give you a lot of advice, a lot of ways to cope with things, a lot of ways to handle things.” (Fiona)  “I know CAMHS is a big one in the country and I know there’s a lot of discrepancy about how good CAMHS is and how bad it is.” (Aco) |
| 3.2 Visuals and professionalism | “It’s laid out in a very professional way, like I said, so I feel like I would trust it in a factual perspective.” (Matilda)  “I think because the NHS they would attract a lot more people so they would just need to put a bit more effort into it, whether it's just pictures, changing the colours a bit, making it more appealing, because there's just the appearance more than anything.” (Sophie)  “The way it's designed, I feel like I mean just with the image just being put in there, it doesn't look like it’s properly done. There's gonna kind of [affect] the credibility because I feel like, I mean, I could do this.” (Jago) |
| 3.3 Resonating with lived experience | “[Sally] may be able to find someone who's [in] a similar boat, or who's been through something similar to her, which means she can get some advice [...] [she’ll] know that she's not the only one that's uh going through that.” (Aco)  “Having like something at like the bottom of the page or a video or something with someone with lived experience as well would make me feel like it's a bit more trustworthy.” (Maisie) |
| ***Theme 4: Help-seeking is a personal journey*** | |
|  | “Under the assumption that this is [Sally’s] first time experiencing, uh, low mood or what[ever] you want to call it, I think it would be a bit overwhelming because there's all these different labels and things that you she doesn't quite-she doesn't-she doesn't know why she feels the way she does yet.” (Aco)  “The advice that they've given is sort of what you'd expect, [it] just say[s] like go for some physical activity, just talk to some people, which doesn't seem too useful if that's what we're getting.” (Flower)  “I'm not the right person for that because I would already be feeling quite hopeless down and not really engaging in it. But as someone maybe who isn't as far and isn't as severe into that process and into that journey, maybe if it was [at] the earliest stages, I would catch it and go, ‘oh, okay, let's-let's give it a go.” (Matilda)  “At some point [this information on medication] may be useful, but for me at this point in [time], initially I don’t think that will be needed.” (Ami) |
